# Protective Effect of Dietary Calcium Intake on Esophageal Cancer Risk: A Meta-Analysis of Observational Studies

**DOI:** 10.3390/nu9050510

**Published:** 2017-05-18

**Authors:** Qianwen Li, Lingling Cui, Yalan Tian, Han Cui, Li Li, Weifeng Dou, Haixia Li, Ling Wang

**Affiliations:** College of Public Health, Zhengzhou University, Zhengzhou 450001, Henan, China; lqw9319@163.com (Q.L.); cuilingling0613@163.com (L.C.); lhk0829@163.com (Y.T.); zhuxic@126.com (H.C.); lili01060209@163.com (L.L.); gaodujuedingshiye@163.com (W.D.); 18538721576@163.com (H.L.)

**Keywords:** esophageal cancer, dietary calcium, meta-analysis

## Abstract

Although several epidemiological studies have investigated the association between dietary calcium intake and the risk of esophageal cancer, the results are inconsistent. This study aimed to make a comprehensive evaluation regarding the association between calcium intake and risk of esophageal cancer through a meta-analysis approach. We searched for all relevant articles from the inception to April 2017, using PUBMED, EMBASE, and Web of Knowledge. The pooled odds ratio (ORs) with the 95% confidence interval (95% CI) for the highest versus the lowest categories of calcium intake was calculated using a Mantel–Haenszel fixed-effect model. In total, 15 articles reporting 17 studies including 3396 esophageal cancer cases and 346,815 controls were selected for the meta-analysis. By comparing the highest vs. the lowest levels of dietary calcium intake, we found that dietary calcium intake was inversely associated with the risk of esophageal cancer (OR = 0.80, 95% CI: 0.71–0.91, *I*^2^ = 33.6%). The subgroup analysis indicated that the protective function of dietary calcium intake were observed in esophageal squamous cell cancer, but not in esophageal adenocarcinoma in the studies conducted in Asia, but not those in Europe and America. In conclusion, our results suggest that higher dietary calcium intake is associated with a lower risk of esophageal cancer—especially esophageal squamous cell cancer—in Asian populations, though more data from prospective cohort studies are needed.

## 1. Introduction

Esophageal cancer (EC) is one of the most common cancers in the world. With estimated 455,800 new cases and 400,200 deaths in 2012, esophageal cancer has been the tenth most common malignancy and the eighth leading cause of cancer-related deaths [1]. In fact, the overall 5-year survival is less than 20%, due to the diagnosis made at advanced stage [2]. Therefore, a better understanding of the etiology is especially important for esophageal cancer prevention and control.

Epidemiologic evidence demonstrated a number of risk factors for esophageal cancer, such as age, gender, alcohol drinking, tobacco smoking, obesity, chronic gastroesophageal reflux disease, dietary carcinogens, and insufficiencies of micronutrient consumption [3,4,5], among which dietary factors may play a more important role [6]. Accordingly, chemopreventive agents for esophageal cancer have attracted great attention, including vitamin C, vitamin E, carotenoids, and various minerals [7,8]. Calcium is an essential element, and is only available to the human body through dietary sources. It plays a critical role in skeletal mineralization, and presents a wide range of biological functions in soft tissues [9], including antitumor properties. Animal studies have suggested that high calcium intake could suppress the cell cycle, promote apoptosis, and reduce the formation of colonic tumors [10]. Compared to oral supplements, dietary calcium intake is relatively safe. Thus, many prior meta-analyses have focused on the association between dietary calcium intake and risk of colorectal cancer, breast cancer, and prostate cancer [11,12,13]. However, there is no systemic analysis carried out regarding the relationship between calcium intake and the risk of esophageal cancer. For esophageal cancer, the published results in epidemiological studies are controversial. Therefore, we conducted a meta-analysis to assess the association of dietary calcium intake and the risk of esophageal cancer.

## 2. Materials and Methods

### 2.1. Search Strategy

Three electronic databases (PUBMED, EMBASE, and Web of Knowledge) were searched aiming to assess the association between dietary calcium intake and esophageal cancer up to April 2017. The following search terms were used: (calcium OR dairy products OR dairy OR milk OR cheese OR yogurt OR cream) AND (esophagus OR esophageal OR oesophageal) AND (cancer OR tumor OR tumour OR carcinoma OR neoplasm). Besides, the references cited within the related articles were also searched for additional eligible publications. Only full-text original journal articles with a cohort or case–control study design were included.

### 2.2. Study Selection

To be included in this meta-analysis, the studies had to meet the following inclusion criteria: (1) the study design was a cohort or case–control study; (2) the exposure of interest was dietary calcium intake; (3) the outcome was esophageal cancer; (4) relative risk (RR), hazard ratio (HR), or odds ratio (OR), and corresponding 95% confidence intervals (95% CI) for the highest versus the lowest calcium intake were reported or could be calculated. If data were duplicated in more than one study, the one with the largest number of cases or the longest follow-up period was selected.

### 2.3. Data Extraction

Two reviewers independently assessed the articles with the inclusion criteria and extracted data with a standardized form [14], and any discrepancies were resolved by a third investigator. Information extracted from each article included the first author’s last name, publication year, country, study design (cohort or case–control study), pathological type (esophageal adenocarcinoma (EAC); esophageal squamous cell carcinoma (ESCC); and mix type, which represents undefined pathological type), numbers of case and control, dietary assessment method, the reported ORs (RRs) and 95% CIs with the most adjustment for the highest versus the lowest calcium intake. Besides, quality assessment was performed according to the Newcastle–Ottawa scale [15]. The scores of 0–3, 4–6, and 7–9 were regarded as low, moderate, and high quality, respectively.

### 2.4. Statistical Analysis

The pooled ORs with corresponding 95% CI (highest versus lowest categories of calcium intake) were calculated to measure the association across studies. The heterogeneity among studies was examined by Q-test and *I*^2^ statistics. Generally, for the Q-test, heterogeneity with a value of *p* < 0.05 was considered as statistically significant. For the *I*^2^ statistic, the following cut-off points were used: <25% (low heterogeneity), 25–50% (moderate heterogeneity), and >75% (severe heterogeneity). If *p* < 0.05 and *I*^2^ > 50%, a DerSimonian and Laird random-effect model was used. Otherwise, a Mantel–Haenszel fixed-effect model was applied [16]. 

Meta-regression and subgroup analyses were performed to explore the possible source of heterogeneity based on geographic location (America, Europe, and Asia), study design (population-based case–control (PBCC), hospital-based case-control (HBCC), and cohort), pathological type (EAC, ESCC, and Mix type), dietary assessment (validated method or not validated method), publication year (before/in 2000 or after 2000), and adjustment for energy intake/body mass index (yes or no) [17]. The “leave-one-out” sensitive analysis was applied to test the stability of the meta-analysis results [18]. Both Begg’s rank correlation test and Egger’s linear regression test were performed to investigate potential publication bias (*p* < 0.10) [19]. The statistical analyses were performed using STATA version 11.0 (Stata Corporation, College Station, TX, USA). All the *p*-values were two-tailed, and *p* < 0.05 was considered as significant, unless explicitly stated.

## 3. Results

### 3.1. Characteristics of the Included Studies

**Figure 1:**
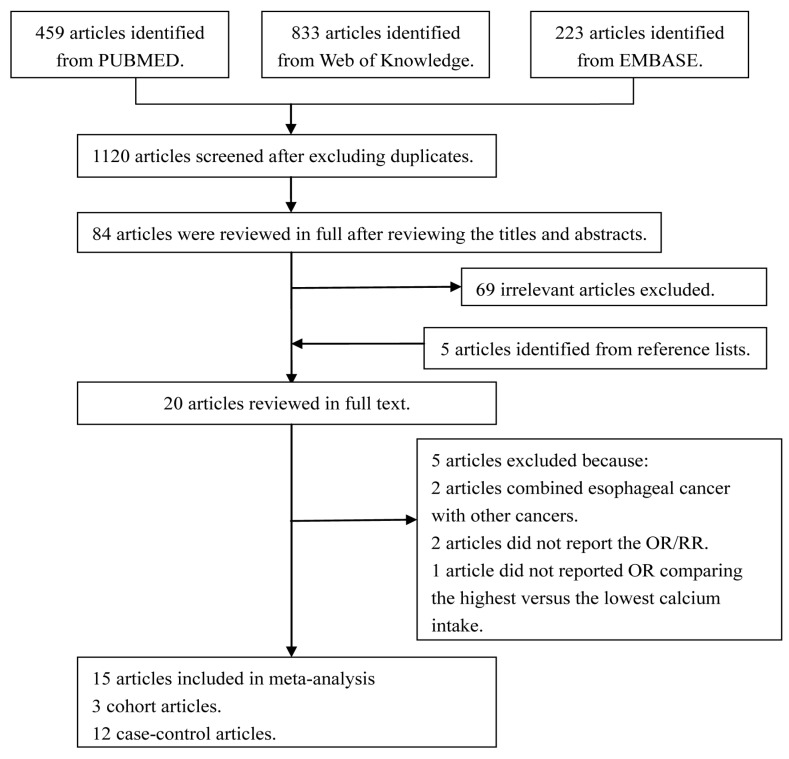
The flow diagram of screened, excluded, and analyzed publications.

The study selection process and the results are shown in Figure 1. With our search strategy, a total of 459 articles from PUBMED, 236 articles from EMBASE, and 833 articles from Web of Knowledge were identified. After removing duplicates and studies that did not meet the inclusion criteria and adding articles identified through references review, 20 articles were reviewed in full. Among them, two articles did not report the OR/RR, two articles reported the association between calcium and cancer of upper aerodigestive tract, and one article only provided the OR comparing the 75th versus the 25th percentile of calcium intake to estimate the risk of esophageal cancer. As a result, 15 articles reporting 17 studies including 3396 esophageal cancer cases and 346,815 controls were selected for the meta-analysis [8,20,21,22,23,24,25,26,27,28,29,30,31,32,33]. Detailed characteristics of the included studies are presented in Table 1.

### 3.2. Meta-Analysis of Calcium Intake and Esophageal Cancer Risk

**Figure 2:**
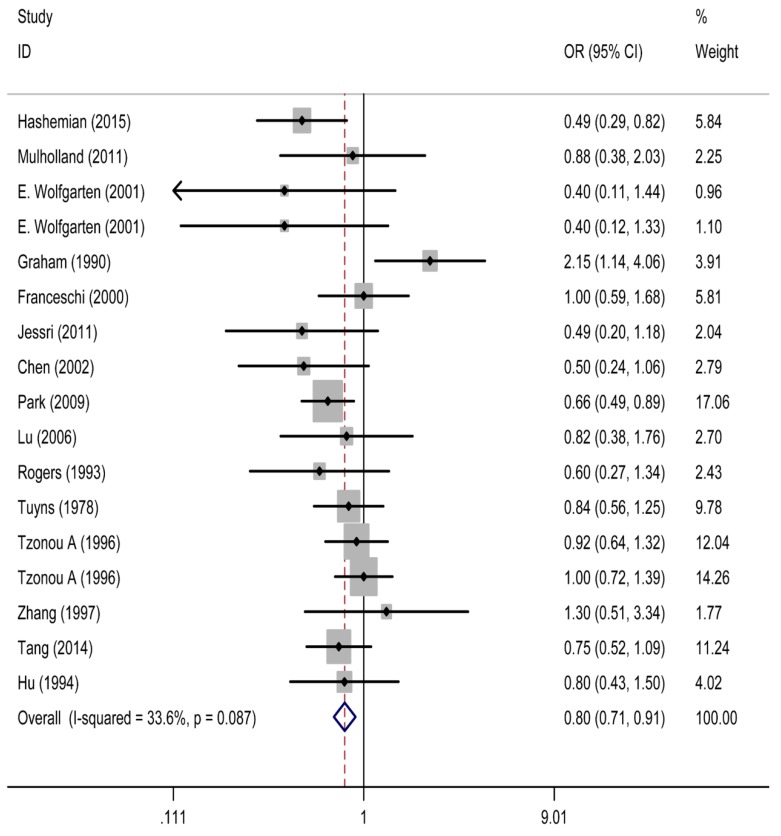
The forest plot between highest vs. lowest categories of dietary calcium intake and esophageal cancer. OR, relative risk; CI, confidence interval.

As shown in Figure 2, the pooled OR of esophageal cancer for the highest versus lowest category of calcium intake was 0.80 (95% CI: 0.71, 0.91), with moderate heterogeneity (*I*^2^ = 33.6%), suggesting that calcium intake was inversely associated with the risk of esophageal cancer.

### 3.3. Heterogeneity Analysis

Next, we conducted meta-regression analysis and subgroup analyses by geographic location, study design, pathological type, dietary assessment, publication year, as well as adjustment for energy intake and BMI. Meta-regression analysis showed that the inverse association between calcium intake and esophageal cancer was not significantly affected by these factors (*p* > 0.05). 

As shown in Table 2, when stratified by geographic location, publication year, and adjustment for energy intake, the statistically inverse association between dietary calcium intake and risk of esophageal cancer remained in studies conducted in Asia (OR = 0.67, 95% CI: 0.52, 0.86, *I*^2^ = 0.0%), published after 2000 (OR = 0.64, 95% CI: 0.53, 0.77, *I*^2^ = 0.0%), and adjusted for dietary energy intake (OR = 0.83, 95% CI: 0.70, 0.98, *I*^2^ = 3.6%), with no statistically significant heterogeneity. Likewise, negative association was also found in studies of ESCC (OR = 0.76, 95% CI: 0.60, 0.96, *I*^2^ = 28.3%), cohort studies (OR = 0.67, 95% CI: 0.54, 0.84, *I*^2^ = 23.6%), and studies with high quality score (OR = 0.76, 95% CI: 0.66, 0.87, *I*^2^ = 12.7%), as well as in studies using validated method (OR = 0.81, 95% CI: 0.48, 1.35, *I*^2^ = 15.5%). The heterogeneity alleviated in all four subgroups. The inverse association was not altered in either subgroup when stratified by adjustment for BMI. Heterogeneity decreased in the subgroup adjusted for BMI, while it increased in the other subgroup.

### 3.4. Sensitivity Analysis and Publication Bias

**Figure 3:**
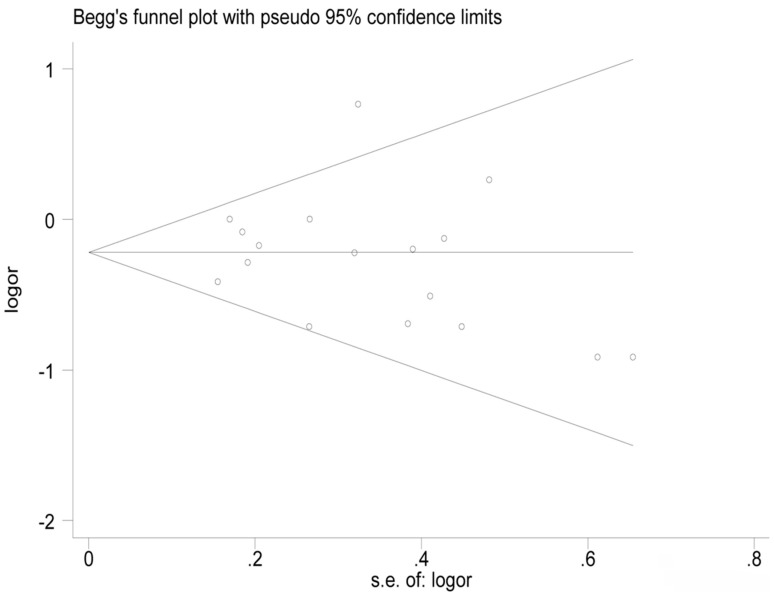
Funnel plots of dietary calcium intake and the risk of esophageal cancer.

Sensitivity analysis was conducted by leaving one study out in turn and pooling the ORs of the remaining studies. The summary ORs did not substantially change, which indicated that our results were statistically robust. However, after excluding the study conducted by Graham [22], there was no statistically significant heterogeneity in the remaining studies (*I*^2^ = 0.0%, *p* = 0.489). Egger’s test showed no evidence of significant publication bias in this meta-analysis (*t* = −0.65, *p* = 0.523). The funnel plot was provided in Figure 3.

## 4. Discussion 

This study is the first systemic meta-analysis regarding the association between dietary calcium intake and the risk of esophageal cancer, which is based on 15 articles reporting 17 studies (3 cohort studies and 14 case–control studies), including 3396 cases and 346,815 controls. The sample size is large enough to evaluate the effect of calcium intake on esophageal cancer.

In addition to its bone formation role, calcium is also a ubiquitous second messenger and plays a critical role in human health [34]. It was hypothesized that calcium intake could reduce the risk of cancer by promoting the activation of transcription factors CREB (cAMP response element binding protein) [35] and oncogenic Ras [36], downregulating the synthesis of 1,25-dihydroxyvitamin D [37], inducing cell cycle arrest, and promoting cell differentiation and tumor cell apoptosis. The previous studies have shown that a high calcium diet could induce cell differentiation and suppress cell proliferation and carcinogenesis production underlying the expression of p120-catenin and the formation of p120-dependent E-cadherin-β-catenin-p120-catenin complex in epithelial tissues in mice [38,39]. In addition, an in vitro model of esophageal squamous cell differentiation proposed that extracellular calcium could induce esophagin expression by upregulating the activity of esophagin promoter, which is silenced at the transcription level in esophageal tumors [40,41]. To date, studies of chemopreventive agents for esophageal cancer have attracted great attention. For example, in our previous meta-analysis, it was found that intakes of anthocyanidins, flavanones, and flavones could significantly reduce the risk of esophageal cancer by 40%, 35%, and 22%, respectively [42]. Likewise, the meta-analysis conducted by Bo and her colleagues showed that vitamin C intake was associated with a 42% reduction in esophageal cancer risk [43]. In the current meta-analysis, we found that calcium intake was associated with a 33% reduction in esophageal cancer risk in Asian populations, which supports another important piece of information for the chemoprevention of esophageal cancer. Numerous studies have investigated the association between dietary calcium intake and various cancer risks. Previous meta-analyses have found that dietary calcium intake might have a protective effect on colorectal cancer (RR = 0.86, 95% CI = 0.78–0.95) [44] and breast cancer (RR = 0.92, 95% CI = 0.85–0.99) [45], but have an opposite influence on the risk of prostate cancer (RR = 1.18, 95% CI = 1.08–1.30) [46], while having no significant relationship with the risk of lung cancer (RR = 0.85, 95% CI = 0.63–1.13) [47]. The findings presented in this meta-analysis add new information regarding the association between calcium intake and cancer risk.

However, some information should be considered in the subgroup analyses. First and foremost, in the subgroup analysis by geographic locations, we found the inverse association between dietary calcium intake and esophageal cancer in studies of Asia, but not in studies of America and Europe, which indicated that the results are acceptable to Asian populations, but cannot be extended to Americans and Europeans. According to the information about calcium intake quantity presented in Table 1, we found that the average of highest dietary calcium intake in Asian populations (621 mg/day) was much lower than in Americans (1284 mg/day) and Europeans (1232 mg/day). Similar findings were reported before [48]. In addition, Dai and colleagues recently reported that high calcium intake may decrease the risk of colorectal adenoma only in the context of the lower dietary calcium: magnesium intake ratio [49]. However, epidemiological evidence reported that the ratio of Ca: Mg intake is much higher in the US (2.8) [50] and Irish populations (3.0) [51] compared with the East Asian population (1.6) [52], which might be another explanation for the heterogeneity in different geographic locations. Second, when stratified by pathological type, a significant inverse association was found in studies of ESCC, but not in studies of EAC, which indicated that the protective effect of dietary calcium intake on esophageal cancer might be pathological type-selective. Third, the inverse association still existed in prospective cohort studies, but not in case–control subgroups, which might be explained by the limitation of sample size and the influence of recall bias in case–control studies. In addition, the inverse association persisted in studies using validated dietary assessment methods for calcium intake estimation, but not in those using non-validated ones. Misclassification of exposure may be introduced, which could lead to an inaccurate estimation of the association between calcium intake and esophageal cancer risk [13]. Moreover, the results also differed when stratified by publication year. We found a strong inverse correlation in studies published after 2000, but no statistical association in studies published before or in 2000. Similarly, we also found an inverse association in studies with high quality scores, but no statistical association in studies with low or moderate quality scores. Thus, we could partially attribute the inconsistent result in studies published before or in 2000 to the lower average quality score (5.9), compared with studies published after 2000 (7.6). Finally, we found that the significant inverse association persisted in studies adjusted by dietary energy intake, but not in the other subgroup, which suggested that the status of dietary energy intake might confound the association between dietary calcium intake and esophageal cancer.

The present meta-analysis has several advantages. First, our analysis is the first comprehensive meta-analysis to reveal the possible associations between dietary calcium intake and risk of esophageal cancer. Second, it enables a reliable conclusion by using meta-regression analyses and sub-group analyses to explore the sources of heterogeneity. However, several limitations should also be noted. First, most of the eligible studies were case–control studies, which were difficult to rule out the influence of recall bias. Second, the levels of dietary calcium intake ranged widely among the studies included in our meta-analysis, and the definitions of the intake categories were different. Another limitation is that this meta-analysis is study-based, but not individual patient-based. Finally, we did not perform a dose–response effect analysis due to the incomplete data of dietary calcium intake. 

## 5. Conclusions

The present study suggests that a higher intake of dietary calcium might have protective effect against esophageal cancer—especially esophageal squamous cell cancer—in Asian populations. To further solidify the association of dietary calcium intake with the risk of esophageal cancer, well-designed studies—especially prospective cohort studies with validated FFQ and adjusted for dietary energy intake—should be conducted.

## Figures and Tables

**Table 1:** Characteristics of the included studies on dietary calcium intake and risk of esophageal cancer.

Author, Year	Country	Study-Design	Pathological Type	Source of Control	Dietary Assessment	Participants (Cases)	Comparison	OR or RR (95% CI)	NOS Score	Adjustment for Covariates
Hashemian, 2015 [8]	Iran	Cohort	ESCC	PB	FFQ-116 items, validated	47,204 (201)	≥1048.0 vs. <409 (mg/day)	0.49 (0.29–0.82)	8	Age, sex, total energy, place of residence, smoking, wealth score, ethnicity, opiate use, BMI, education, marital status, physical activity score, and fruit and vegetable intakes
Mulholland, 2011 [20]	Ireland	Case–control	EAC	PB	FFQ-101 items, validated	252 (218)	≥1262.0 vs. <929.3 (mg/day)	0.88 (0.38–2.03)	6	Age, sex, energy intake, smoking status, BMI, education, occupation, alcohol, regular non-steroidal anti-inflammatory drug use, *Helicobacter pylori* infection, energy-adjusted glycemic index intake, energy-adjusted saturated fat intake, and location
Wolfgarten, 2001 [21]	Germany	Case–control	EAC	PB	DHQ, NA	100 (40)	>1590 vs. <986 (mg/day)	0.4 (0.1–1.3)	7	Age, residence, and nationality
Wolfgarten, 2001 [21]	Germany	Case–control	ESCC	PB	DHQ, NA	100 (45)	>1590 vs. <986 (mg/day)	0.4 (0.1–1.1)	7	Age, residence, and nationality
Graham, 1990 [22]	United states	Case–control	Mix type	PB	FFQ, NA	174 (178)	>1028.5 vs. <543.5 (mg/day)	2.15 (1.14–4.06)	4	Sex, age, education, smoking, and alcohol ingestion
Franceschi, 2000 [23]	Italy	Case–control	ESCC	HB	FFQ-78 items, validated	743 (304)	Q5 vs. Q1	1.0 (0.6–1.7)	8	Age, gender, area of residence, education, physical activity, BMI, tobacco smoking, alcohol drinking, and non-alcohol energy
Jessri, 2011 [24]	Iran	Case–control	ESCC	HB	FFQ-125 items, validated	96 (47)	T3 vs. T1	0.49 (0.15–0.87)	7	Age, sex, gastroesophageal reflux disease symptoms, BMI, smoking status, smoking intensity and duration (pack-years), physical activity, and education level
Chen, 2002 [25]	United states	Case–control	EAC	PB	DHQ, validated	449 (124)	Q4 vs. Q1	0.5 (0.2–0.9)	7	Age, age squared, gender, respondent type, BMI, alcohol use, tobacco use, education level, family history of respective cancers, and vitamin supplement use
Park, 2009 [26]	United states	Cohort	Mix type	PB	FFQ-124 items, validated	29,3439 (468)	>1247 vs. <478 (mg/day)	0.66 (0.49–0.90)	9	Smoking status, time since quitting smoking, smoking dose, antacid use, personal history of diabetes, and hypertension
Lu, 2006 [27]	China	Case–control	ESCC	PB	FFQ-97 items, NA	415 (218)	≥344 vs. <157 (mg/day)	0.82 (0.38–1.75)	8	Age, gender, educational level, income, BMI, total energy intake, smoking, and drinking.
Rogers, 1993 [32]	United states	Case–control	Mix type	PB	FFQ, NA	593 (127)	>1419 vs. <571 (mg/day)	0.6 (0.3–1.5)	6	Age, sex, pack-years of cigarette use, drink-years of alcohol, energy intake, β-carotene intake, and ascorbic acid intake
Tuyns, 1987 [28]	France	Cohort	mix type	PB	DHQ, validate	2788 (743)	>1000 vs. <600 (mg/day)	0.84 (0.56–1.25)	3	Age, alcohol consumption, and tobacco smoking
Tzonou A 1996 [31]	Greece	Case–control	ESCC	HB	FFQ-115 items, validated	243 (43)	Q5 vs. Q1	0.92 (0.64–1.32)	7	Gender, age, birthplace, schooling, height, analgesics, coffee drinking, alcohol intake, tobacco smoking, and energy intake.
Tzonou A 1996 [31]	Greece	Case–control	EAC	HB	FFQ-115 items, validated	256 (56)	Q5 vs. Q1	1 (0.72–1.4)	7	Gender, age, birthplace, schooling, height, analgesics, coffee drinking, alcohol intake, tobacco smoking, and energy intake, though not mutually
Zhang, 1997 [29]	United States	Case–control	EAC	HB	HHHQ, validated	189 (29)	Q4 vs. Q1	1.3 (0.5–3.3)	5	Age, sex, race, education, total dietary intake of calories. Smoking, alcohol use, and BMI
Tang, 2014 [30]	China	Case–control	mix type	HB	FFQ-137 items, validated	739 (359)	>470 vs. <260 (mg/day)	0.75 (0.52–1.1)	8	Age, gender, education level, BMI, total energy intake, smoking status, alcohol drinking, and family history of cancer in first-degree relatives.
Hu,1994 [33]	China	Case–control	mix type	HB	FFQ-32 items, NA	588 (196)	Q4 vs. Q1	0.8 (0.4–1.4)	7	Alcohol intake, smoking. and family income

Abbreviations: EAC, esophageal adenocarcinoma; ESCC, esophageal squamous cell cancer; HB, hospital-based; PB, population-based; DHQ, Dietary History Questionnaire; FFQ, Food Frequency Questionnaire; HHHQ, Health Habits and History Questionnaire; OR, odds ratio; CI, confidence interval; N/A, not available.

**Table 2:** Subgroup analysis of dietary calcium intake and risk of esophageal cancer.

Subgroups	No. of Studies	No. of Cases	Pooled ORs (95% CI)	*p*	Heterogeneity Test		
					Chi-Square	*I*^2^	P_het_
**All studies**	17	3396	0.80 (0.71, 0.91)	0.001	24.11	33.6%	0.087
**Location**							
Europe	7	1449	0.90 (0.75, 1.08)	0.262	3.97	0.0%	0.681
America	5	926	0.88 (0.51, 1.49)	0.625	13.83	71.1%	0.008
Asia	5	1021	0.67 (0.52, 0.86)	0.002	2.80	0.0%	0.591
**Study design**							
Cohort	3	1412	0.67 (0.54, 0.84)	0.000	2.62	23.6%	0.270
PBCC	7	950	0.84 (0.61, 1.14)	0.261	13.68	56.2%	0.033
HBCC	7	1034	0.89 (0.74, 1.06)	0.182	4.00	0.0%	0.677
**Pathological type**							
ESCC	6	858	0.76 (0.60, 0.96)	0.019	6.97	28.3%	0.223
EAC	5	467	0.89 (0.68, 1.16)	0.381	4.84	17.4%	0.304
Mixed type	6	2071	0.84 (0.63, 1.13)	0.252	11.50	56.5%	0.042
**Dietary assessment**							
Validated method	11	2592	0.79 (0.69, 0.90)	0.001	11.84	15.5%	0.296
Not Validated method	6	804	0.81 (0.48, 1.35)	0.413	11.68	57.2%	0.039
**NOS score**							
Low quality	1	743	0.84 (0.56, 1.25)	0.395	0.00	N/A	N/A
Moderate quality	4	552	1.14 (0.63, 2.05)	0.671	6.64	54.8%	0.084
High quality	12	2101	0.76 (0.66, 0.87)	0.000	12.60	12.7%	0.320
**Adjustment for energy intake**							
Yes	8	1251	0.83 (0.70, 0.98)	0.031	7.26	3.6%	0.402
No	9	2145	0.78 (0.58, 1.04)	0.093	16.61	51.8%	0.034
**Adjustment forBMI**							
Yes	8	1500	0.72 (0.58, 0.90)	0.003	7.16	2.2%	0.413
No	9	1896	0.85 (0.73, 0.99)	0.037	15.57	48.6%	0.049
**Publication year**							
Before/in 2000	8	1676	0.97 (0.82, 1.16)	0.767	8.75	20.0%	0.271
After 2000	9	1720	0.64 (0.53, 0.77)	0.000	4.58	0.0%	0.802

Abbreviations: ESCC, esophageal squamous cell cancer; EAC, esophageal adenocarcinoma; PBCC, population-based case–control; HBCC, hospital-based case–control; NOS, Newcastle–Ottawa scale; BMI, body mass index; OR, odds ratio; CI, confidence interval; N/A, not available.
